# Identification and characterization of new Sp1 sites located in the R region of the Human T-lymphotropic virus 1 (HTLV-1) long terminal repeat

**DOI:** 10.1186/1742-4690-11-S1-P108

**Published:** 2014-01-07

**Authors:** Benoit Van Driessche, Gwenaëlle Robette, Caroline Vanhulle, Arsène Burny, Olivier Rohr, Carine Van Lint

**Affiliations:** 1 Laboratory of Molecular Virology, Institut de Biologie et de Médecine Moléculaires (IBMM), Université Libre de Bruxelles (ULB), Gosselies, Belgium; 2 Institut Universitaire de Technologie Louis Pasteur de Schiltigheim, University of Strasbourg, Schiltigheim, France

## 

HTLV-1 infection is characterized by viral latency in the large majority of infected cells and by the absence of viremia. These features are thought to be due to the transcriptional repression of viral expression in vivo. Specific protein 1 (Sp1) binds to more than 1000 different cellular promoters and regulates the expression of numerous genes involved in cell proliferation, apoptosis, and differentiation. In silico analysis of the nucleotide sequence of the HTLV-1 LTR revealed the presence of two new potential Sp1 binding sites within the R region. We demonstrated that the Sp1 and Sp3 transcription factors bound in vitro to these sites by EMSAs and supershift experiments. We also performed competition assays with a probe corresponding to a Sp1 binding site consensus in order to compare Sp1 binding affinity for the four previously reported and the two newly identified Sp1-binding sites located in the HTLV-1 promoter. Point mutations of the known and newly identified Sp1 sites were introduced in the HTLV-I LTR cloned in either the sense or the antisense orientation in the context of an episomal reporter vector. We demonstrated that the Sp1 sites interfered with both the sense transcription from the 5’LTR and the antisense transcription from the 3’LTR (necessary for HBZ expression in vivo). ChIP experiments to evaluate in vivo Sp1 binding to the HTLV-I LTR U3 region are currently under investigation. Our results demonstrate the presence of two new functional Sp1 binding sites located in the R region of the HTLV-I LTR.

